# Dimensional phenotype measurement in children with rare genetic conditions: new insights into the aetiology of neurodevelopmental and psychiatric disorders

**DOI:** 10.3389/fpsyt.2026.1602131

**Published:** 2026-04-13

**Authors:** SJRA Chawner, LC Chetcuti, EK Spackman, M Uljarevic

**Affiliations:** 1 Centre for Neuropsychiatric Genetics and Genomics, Cardiff University, Cardiff, United Kingdom; 2 Yale Child Center, Yale University, New Haven, CT, United States; 3 Department of Psychiatry and Behavioral Sciences, Stanford University, Stanford, CA, United States

**Keywords:** child and adolescent psychiatry, dimensional assessment, genomics, HiTOP, medical genetics, psychiatric genetics, rare genetic conditions, RDoC

## Abstract

Rare neurodevelopmental genetic conditions (NGCs) present with diverse and complex phenotypic manifestations, often resulting in a range of clinical and cognitive difficulties that cut across traditional, categorically defined neurodevelopmental and neuropsychiatric diagnoses. Traditional categorical diagnostic frameworks have significant limitations in capturing the full complexity and heterogeneity of these phenotypes. This perspective reviews current advances in the field, highlighting the benefits of dimensional frameworks like the Research Domain Criteria (RDoC) and the Hierarchical Taxonomy of Psychopathology (HiTOP). Dimensional approaches have shown promise in capturing subthreshold symptoms and behavioural dimensions predictive of later neuropsychiatric outcomes. The transition to dimensional frameworks offers significant potential for improving diagnostic accuracy and informing personalised treatment strategies. However, the field remains hindered by the lack of standardised and validated dimensional assessment tools. Future research should focus on developing new assessment tools that are specifically designed for NGCs and are culturally and neurodivergence sensitive, with researchers, clinicians, and families codeveloping measures to ensure the practical application of these tools.

## Introduction

The increasing number of children diagnosed with rare neurodevelopmental genetic conditions (NGCs), including structural genomic variants such as copy number variants (CNVs), and sequence-level variants such as single nucleotide variants (SNVs), underscores the urgent need to understand their neurodevelopmental and neuropsychiatric outcomes.

NGCs present with complex and diverse phenotypic manifestations, including variable cognitive phenotype ranging from severe intellectual disability to normative cognitive functioning, autism, attention-deficit/hyperactivity disorder (ADHD), and psychiatric conditions such as schizophrenia, anxiety and mood disorders, with multi-morbidity being rule rather than exception (1–8). To truly deliver tailored care approaches for children with rare neurodevelopmental genetic conditions, there is a strong need for research that captures the complexity of these phenotypes and their underlying mechanisms.

In this perspective, we provide an overview of the limitations that the traditional categorical approaches have posed for advancing the understanding of the neurodevelopmental and neuropsychiatric phenotypes in NGCs, summarise current dimensional alternatives and highlight their benefits across research and clinical contexts and outline strategies to advance research and clinical practice.

## Neurodevelopmental and neuropsychiatric presentations in NGCs: current state of the field

Both clinical and population based studies document high rates of neurodevelopmental (NDD) and neuropsychiatric (NPD) diagnoses^1^ in individuals with NGCs (1, 9). However, a limitation of traditional categorical diagnostic frameworks is that they fail to capture the full phenotypic complexity of these conditions, including subthreshold psychopathology, transdiagnostic impairments, and developmental risk indicators of later emergence of psychiatric conditions (10–16). To exemplify, in a study of autism in neurodevelopmental CNVs, 54% of individuals with one of the four CNVs who did not meet full autism diagnostic criteria had elevated levels of autistic traits that met clinical cut-offs (17). Furthermore, several studies using dimensional measures have identified a range of cognitive and behavioural developmental predictors of emergence of psychotic phenomena in 22q11.2 Deletion Syndrome (11, 18, 19).

Large-scale collaborative efforts, such as the International Consortium on Brain and Behaviour in 22q11.2 Deletion Syndrome (20) and the Simons Searchlight (previously Simons Variation in People) have been established to examine genotype-phenotype relationships at scale (21). In particular, these previously unavailable samples have significantly advanced our understanding of the genetic and phenotypic variability of NGCs, including detailed phenotypic descriptions of CNVs at the 22q11.2 and 16p11.2 loci, combining data from international clinical sites (22–24). However, despite the unprecedented sample size and a broad range of assessments available within these studies, most quantify neurodevelopmental and neuropsychiatric challenges by means of categorical diagnostic status, which do not adequately capture the dimensional nature of NDD and NPD traits. Thus, to facilitate clinical characterisation and understanding of mechanisms underpinning diverse clinical and cognitive presentations across NGCs, and to ultimately inform the development of effective and individually tailored pharmacological and psychosocial treatment approaches, it is essential to transition toward the developmental transdiagnostic frameworks that have recently emerged as promising alternatives for addressing challenges of the current categorical approaches.

## An overview of research to date that has used dimensional approaches with children with rare chromosomal conditions

The relationship between NGCs and phenotypic outcome is complex. Variants do not align to discrete neuropsychiatric phenotypes; rather, neuropsychiatric phenotypic outcomes are overlapping. Findings from the IMAGINE-ID cohort illustrate this with 13 rare neurodevelopmental CNVs impacting core NDDxdimensional traits and having broader differential effects on co-occurring cognitive and mental health features (8). Although rare variants did not align to discrete neuropsychiatric presentations, subtle qualitative (neuropsychiatric domain) and quantitative (dimensional score severity) differences were present. Previous authors have characterised the genotype-phenotype relationships as “all for one, and one for all”, in that one rare variant (e.g. 22q11.2 Deletion Syndrome) is associated with multiple NPD and NDD phenotypes, and conversely, one NPD or NDD trait (e.g. ADHD traits) is associated with multiple rare variants (25).

Genetics does not carve psychiatric classifications at its joints; rather, findings from studies of psychiatric-risk rare variants highlight the biological overlap between neurodevelopmental conditions and adult psychiatric outcomes, including depression and schizophrenia (2, 3, 26, 27). As such, dimensional approaches to NGCs provide a unique opportunity to identify constructs that better align with biological risk and, therefore, may be more amenable to treatment. A key step towards facilitating these approaches is the development of large-scale data consortiums, such as the Genes to Mental Health Network (G2MH) which represents a funded National Institute of Mental Health initiative that is collecting and analysing large-scale data sets combining genomics and dimensional measures of psychopathology spanning diverse populations and geographical regions (16).

A range of studies have documented that individuals with NGCs often experience certain NDD and NPD symptoms that, whilst sometimes below traditional diagnostic thresholds, have significant negative impacts on their concurrent functioning and long-term outcomes. For example, in the Icelandic deCODE population cohort it was found that psychiatric risk CNVs in individuals without a psychiatric diagnosis nonetheless resulted in subtle cognitive deficits (14). The IMAGINE-ID study of children with psychiatric risk CNVs and SNVs identified via UK medical genetic clinics, reports a range of differences in dimensional measures of cognition and neurodevelopment, including motor, attention, social functioning and peer relationships.

Dimensional approaches as well as important for capturing behavioural and cognitive changes which are clinically subthreshold, they also capture traits which may be developmentally predictive of later psychiatric outcomes (8). For example, sleep symptomatology has been identified as a transdiagnostic predictor of mental health outcomes in children with NGCs (28). Several prospective longitudinal studies of children and young people with 22q11.2 Deletion Syndrome, who are at high risk of schizophrenia in adulthood, have identified dimensional measures of cognition and psychopathology that predict later emergence of psychosis (11, 18, 19). An international longitudinal study by the IBBC found that the VIQ trajectory of those children who later developed psychosis diverged from age 11 (12).

Despite emerging work highlighting the promise of dimensional measures, the limitations of available instruments have limited progress and integration of dimensional frameworks across clinical and research contexts.

## Dimensional alternatives to current categorical approaches

The limitations of categorical diagnosis, including pronounced within-diagnosis heterogeneity, high rates of co-morbidity, lack of empirical and factorial validity of the diagnostic phenotypes, and a high degree of shared aetiological factors among supposedly distinct conditions, present significant obstacles across research and clinical contexts. These limitations of categorical diagnosis hinder comprehensive assessment of clinical, educational and developmental need and ultimately, and serve as barriers to delivering prospective early intervention approaches for affected individuals and their families. In response, to address the described limitations at the heart of the traditional categorical systems, the field has increasingly embraced dimensional alternatives, with the NIMH’s Research Domain Criteria (RDoC) and the Hierarchical Taxonomy of Psychopathology (HiTOP) (29) being the most prominent and well-studied frameworks.

In contrast to the DSM-5 and ICD-11, which base psychiatric diagnoses on presenting signs and symptoms, RDoC seeks to ground psychiatric classification in a scientifically supported model of fundamental biobehavioural dimensions that transcend current disorder boundaries. HiTOP aims to establish an empirically based clinical classification system rooted in the natural covariance structure of signs, symptoms, maladaptive behaviours, and traits. While HiTOP, like the DSM-5 and ICD-11, focuses on symptoms and observable traits, it is more firmly rooted in a data-driven approach. Specifically, by utilising the co-variance among the symptoms occurring across different categorically based disorders to reduce the high-dimensional phenotype space, HiTOP aims to arrive at a more parsimonious solution to the structure of psychopathology that is more conducive to neurobiological investigations and identification of specific treatment targets. Furthermore, HiTOP organises clinical constructs into a multilevel hierarchical structure, ranging from narrow and specific clusters of features to broader, more general spectra of interrelated diagnostic phenomena, with a general factor of psychopathology at the highest level. By organising symptoms and traits across different levels of a hierarchical structure, HiTOP allows for studying etiological mechanisms at multiple levels - such as neurobiological mechanisms operating distinctly at the level of specific symptoms, clusters of related traits, or broader spectra.

RDoC provides an important synergy to the HiTOP framework, by putting forward distinct, biologically grounded processes that can be linked to the specific symptom presentations across the hierarchical structure derived by the HiTOP framework. The RDoC framework has put forward fundamental processes, including negative and positive valence, cognitive ability, social processes, and arousal that encompass more specific constructs and subconstructs, each of which is governed by at least partially distinct neural systems, that govern one’s ability to function across different contexts, and that if impaired, can result in diverse clinical presentations seen across NDD and NPD. Together, by defining the structure of the clinical phenotype and putting forward neurobiologically valid and well-defined potential mechanisms, HiTOP and RDoC provide a testable framework for characterising the relationships between narrowly defined, biologically meaningful processes and more granular, data-driven clinical phenotypes. Ultimately, by distinguishing mechanisms that represent broader risk across multiple clinical phenotypes from those that underpin more specific symptom expressions, the described approach can facilitate the development and evaluation of more effective and personalised interventions, addressing both the specific needs and the broader, interrelated dimensions of the phenotype.

Dimensional assessments show great potential to be implemented in routine clinical settings. Clinicians can adopt a stepwise approach, starting with brief screening of higher-order domains using short, self-report instruments that already have normative data and then cascade to more detailed assessments only when time and resources permit. This mirrors the “review of systems” approach in general medicine and allows prioritisation of urgent concerns (e.g., suicidality) without requiring exhaustive evaluation (30). Importantly, dimensional scores can be converted into standardised scores and grouped into pragmatic ranges (e.g., mild, moderate, severe), similar to blood pressure cutoffs, to guide stepped-care decisions. In low-resource settings, feasibility can be enhanced through computerised adaptive testing and smartphone apps. As part of the IMAGINE-ID study of rare neurodevelopmental genetic variants, caregivers completed online versions of the Strengths and Difficulties Questionnaire (SDQ) (31) on 2397 individuals, highlighting the potential to integrate online assessments into clinical practice (1).

Below, we first summarise the current obstacles that have hindered the full adoption of these frameworks in NGC research.

## Limitations of the current assessments

Utilisation of dimensional frameworks such as the HiTOP and the RDoC across research and clinical contexts is heavily reliant on the availability of appropriate assessments. As emphasised by the Workgroup on Tasks and Measures for RDoC report, fit-for-purpose measures are essential for enabling and accelerating the adoption of the RDoC framework into research and clinical practice. Unfortunately, given the fact that both HiTOP and RDoC have emerged relatively recently, very few instruments have been specifically developed to provide sufficient domain representation and coverage that is aligned with either framework (32, 33).

One approach to overcome the lack of bespoke RDoC measures has been to conduct single-instrument and multi-instrument factor analytic studies to appraise the ability of existing general quantitative severity instruments to approximate certain RDoC constructs and subconstructs (34, 35).To exemplify, in a sample of *n* = 27,953 individuals (*M_age_* = 9.55 years, *SD* = 3.79) spanning normative (33.8%) and clinical populations (66.2%), exploratory structural equation modelling (ESEM) and confirmatory factor analysis (CFA) was used to test the ability of the Social Responsiveness Scale (SRS-2) and the Social Communication Questionnaire (SCQ) to depict specific RDoC social processes. Attachment and affiliation and production of facial and non-facial communication constructs emerged across both the SRS-2 and SCQ, and understanding mental states construct emerged from the SRS-2. Nevertheless, item-level analyses revealed significant limitations of both measures for capturing important RDoC social constructs. Therefore, while general quantitative severity instruments are limited for capturing dimensional variation within the RDoC framework, measures that isolate patterns of strengths and challenges within distinct areas of social functioning are, in general, well aligned with specific RDoC constructs. Further, the RDoC framework presents a range of observational and experimental paradigms that involve direct assessment of constructs and encourages a multi-modal measurement approach, including the integration of questionnaire data with observational and experimental paradigms, in order to capitalize on the unique information yielded by each methodology. Parent-report and direct child assessments of a trait may not always correlate highly (36).

The noted measurement limitations not only hinder the adoption of the RDoC-aligned measurement approach but also pose challenges to the broader adoption of the HiTOP framework. More specifically, although a number of informant-report measures, including the Child Behaviour Checklist (CBCL) (37), the Behaviour Assessment System for Children, 3rd Edition (BASC-3) (38), Aberrant Behaviour Checklist (ABC) (38, 39) and the SDQ (31), are available for capturing a broad range of symptoms and higher-order psychopathological dimensions in youth, none of these widely used comprehensive instrument screeners isolates more specific symptom dimensions and clinical presentations (such as DSM-5-defined anxiety subtypes), nor several clinical domains that are important for NDD, including motor functioning and sleep problems. This is a significant limitation given that distinct clinical subdomains tend to show different trajectories and associations with other aspects of clinical phenotype, and, crucially, might respond to different treatments.

Further to the described construct coverage and domain representation limitations, with rare exceptions, existing measures lack evidence for invariance along the normative-psychopathology continuum and across relevant cognitive and demographic variables, including sex, age, IQ, race, and ethnicity. Measurement invariance evaluates the psychometric equivalence of a measurement construct across various groupings. Specifically, measurement invariance is present when a particular construct measures similarly across groups, having a similar structure and meaning to those groups (40). It is therefore critical to ensure that observed differences in groups are not due to measurement bias, as scores resulting from non-invariant scales across target groups are not comparable. Thus, at present, the validity of the use of described assessments in a true dimensional fashion is under question.

In addition to the highlighted general limitations of the existing instruments, it is essential to emphasise specific limitations in the context of the NGCs. More specifically, the majority of the most widely used measures have not been developed specifically for individuals with NGCs who show variable and complex profiles of co-occurring symptomatology that present in an atypical manner. For instance, it is well established that individuals with NDD, in particular autism, that show cognitive profiles similar to those seen across NGCs might present with idiosyncratic anxiety and depression symptoms, including unusual specific phobias (e.g., vacuum cleaners, toilets), fears of change or novelty (41–44), and depression expressed through the reduction in restricted interests. Furthermore, general instruments contain a number of items that capture symptoms whose expression is dependent on the verbal ability of the child/adolescent, thus limiting their utility and applicability to populations with intellectual disability who have low verbal ability or are non-verbal.

A further important issue to consider is the overlap between the core symptoms of several NDD and NPD that are commonly observed in NGCs, such as between core symptoms of autism and those attributed to anxiety and depression. For instance, while the lack of social approach and engagement can be considered a symptom of social phobia, it can also be a manifestation of the low social motivation that characterises autism. Thus, specific symptoms, without the additional contextualisation, can be either interpreted as a part of the core autism symptomatology when they are part of other NDD or NPD or, conversely, could be wrongly attributed to other disorders. Within the 22q11.2 Deletion Syndrome field this has resulted in an ongoing debate whether autism measures that suggest high autistic traits in childhood are, in fact, indexing a prodromal psychotic phenomena rather than autistic traits (45, 46).

Many dimensional assessments such as IQ tests may not be the most suitable for assessing individuals with rare NGCs who are likely to experience cognitive impairments, and therefore more likely to perform at the floor of the range that these measures assess (47). The majority of dimensional measures are designed to optimally capture the mean performance of the population, whereas they are less reliable at population extremes. It also leads to missing data, for instance in a study of cognition in 16p11.2 deletion and duplication carriers, 11% of deletion carriers and 27% of duplication carriers were unable to perform tasks (48). There is a need to increase inclusivity of dimensional assessments, so they meet the complex needs of children with rare NGCs.

## Recommendations for future research and the development of new measurement tools

Given the described limitations of the current assessment landscape for capitalising on the opportunities presented by the dimensional approaches such as RDoC and HiTOP for facilitating our understanding of NGCs, it is clear that urgent changes are needed. There are two approaches to addressing the current measurement limitations: (i) utilizing state-of-the-art psychometric approaches to improve the performance and maximize the utility of the current measures for the dimensional approach, and (ii) developing new measures.

Despite noted limitations of current instruments, integrative data analysis framework and advanced latent variable modelling approaches present an opportunity to capture important RDoC constructs through existing measures, albeit in a somewhat imprecise way (34, 35, 49). More specifically, multi-instrument approaches offer the potential to capture constructs beyond the capabilities of standard single-instrument scoring. For instance, by applying multi-instrument exploratory structural equation modelling to items from a range of different caregiver-report questionnaires, a recent study by Chetcuti et al. was able to derive three distinct processes underpinning social withdrawal in a diverse transdiagnostic sample spanning a range of NDD and NPD (50). To further illustrate the utility of this approach for characterizing heterogeneity in these processes, the authors used derived multi-instrument factor scores in latent profile analyses to identify four distinct profiles of functioning across social ‘reticence’, ‘seeking’, and ‘maintaining’ components, illustrating the utility of focusing on specific, well-defined constructs for understanding clinical heterogeneity (50). Therefore, utilising advanced latent variable approaches to optimize current instruments to approximate relevant dimensional constructs can provide an important bridge toward initial testing of the explanatory power of the dimensional frameworks.

Building on the work by Chetcuti et al., we illustrate the potential utility of capturing more distinct facets and processes linked to overall clinical phenomena that are traditionally thought of and researched within the confines of the categorically defined diagnoses and focus on the opposite ends of the social drive that are traditionally reported among individuals with 22q11.2 Deletion Syndrome and Down Syndrome. While the prevalence of autism, psychoses, and social anxiety, each of which is associated with reduced social drive, is high among individuals with 22q11.2 Deletion Syndrome, an active social drive has been commonly highlighted in individuals with Down Syndrome. However, as noted above, there is pronounced heterogeneity with regard to the social drive among individuals with idiopathic autism (34, 50), and similarly, there are pronounced differences with regard to the lack of social drive among individuals with non-NGCs related schizophrenia. In addition, more recent studies have highlighted wide individual differences with respect to social drive among individuals with Down Syndrome, ranging from indiscriminate social approach to social disinterest and active social avoidance e.g (51, 52).

From both a mechanistic and treatment-related perspective, it is clear that adopting an individual differences approach is essential in order to identify subgroups within 22q11.2 Deletion Syndrome and Down Syndrome with regard to their individual differences in social drive. However, across previous research, studies have relied on disorder-specific conceptualizations and measurement of the atypicalities in social drive, formalizing it as social motivation in autism and social anhedonia in psychosis, with both overlaps and distinctions across constructs and their constituting component, creating a considerable challenge for generalizing findings and understanding specific mechanisms underpinning these difficulties. For instance, without the additional contextualisation, it is unclear whether the lack of social engagement observed among individuals with 22q11.2 Deletion Syndrome should be attributed to the presence or risk for autism or schizophrenia and similarly, whether the low social drive in Down Syndrome should be attributed to low social motivation or social anxiety. To address this lack of conceptual clarity that can drive important differences in how social drive manifests and the specific processes underpinning these different manifestations, we draw on animal and human reward processing literature that has identified fundamental processes underlying motivated behaviour across social and nonsocial contexts have recently proposed a model that deconstructs social drive into ‘orienting’, ‘wanting’, ‘pursuing’, ‘liking’ and ‘learning’ (See **Table 1** for definitions of each process and underlying neural substrates). By utilizing this model, research can go beyond broad, disorder-specific descriptors of the multifaceted social drive difficulties and thus advance mechanistic and phenomenological understanding of this clinically impactful phenomenon in a way that is aligned with the dimensional principles and can lend itself to informing individually tailored treatment approaches. In particular, following the RDoC methodological approach, it will be crucial to investigate potential alterations in genetic, circuit-based and neurocognitive units of analysis and their interactions (53).

**Table 1:** Five processes underpinning social drive.

Component	Definition	Neural substrates
Orienting	Detection of social stimuli and activation of interest or desire.	Anterior Cingulate Cortex; Orbitofrontal Cortex; Amygdala; Hippocampus; Thalamus; Fusiform Face Area; Posterior Superior Temporal Sulcus; Occipital Face Area.
Wanting	Arousal in anticipation of action or reward. Aligns with the anticipatory phase of reward processing.	Ventral striatum (Nucleus Accumbens); Ventral Tegmental Area; Posterior Cingulate Corex; Angular Gyrus; Inferior Parietal Lobule; Medial Prefrontal Cortex.
Pursuing	Decision-making influenced by the consideration of potential benefits relative to cognitive, affective, and physical costs, such as effort and time.	Anterior Cingulate Cortex; Orbitofrontal Cortex; Ventral striatum (Nucleus Accumbens); Ventromedial Prefrontal Cortex; Supplemental Motor Area.
Liking	Immediate pleasure or enjoyment experienced in response to social stimuli. Aligns with the consummatory phase of reward processing.	Amygdala; striatum (Nucleus Accumbens); Ventromedial Prefrontal Cortex; Hippocampus; Brainsteam; Ventromedial Prefrontal Cortex; Temporal Pole.
Learning	Updating of stimulus-reward associations based on feedback.	Orbitofrontal Cortex; Ventral striatum; Hippocampus; Ventromedial Prefrontal Cortex.

Based on Chetcuti et al. (49).

Despite the importance of optimising current instruments through the process described above, their considerable limitations, both in terms of alignment with dimensional models and the applicability to NGCs, necessitate the need for comprehensive measures that are stringently validated and standardised. An important positive recent example is the development of the Neurobehavioral Evaluation Tool (NET) (54, 55), a set of 11 informant-report questionnaires and 12 webcam-collected patient performance measures developed and preliminarily validated in NGCs. The NET was developed based on the recommendations for item generation and refinement outlined by the National Institute of Health Scientific Standards of the Patient-Reported Outcomes Measurement Information System (PROMIS). The development process involved concept elicitation interviews, cognitive interviewing, and quantitative assessments of applicability, readability, and relevance to a range of NGCs. For informant-report, psychometric evaluation demonstrated strong factor structure, very good model (ω = 0.83–0.99) and internal consistency reliability (*α* = 0.82–0.98) as well as excellent test–retest reproducibility at 1-month follow-up (*r* = 0.78–0.98). For the webcam-collected tasks, valid completion rates were high (above 87% across measures) and across all tasks, there was excellent internal consistency reliability (*α* = 0.67 to 0.95) and fair to good 1-month test–retest reproducibility (*r* = 0.40–0.73).

International phenotyping efforts are crucial for providing large, powered cohorts for research, however, there is a strong need for dimensional assessments that are culturally sensitive (16). The NeuroDev study of genotype-phenotype relationships within neurodevelopmental conditions, has adapted measures for Kenyan and South African populations (56). For example for the Molteno neurodevelopmental assessment “child has a tricycle at home” was changed into “child can walk on his/her tiptoes” along with the altered administration of a few fine-motor related items (57).

## Conclusion

This perspective highlights the significant limitations of traditional categorical diagnostic frameworks in capturing the complexity of neurodevelopmental and neuropsychiatric phenotypes in children with NGCs and the importance of leveraging dimensional frameworks to explore the underlying mechanisms of NGCs. This includes identifying specific neurobiological processes that contribute to the development of neuropsychiatric symptoms. A limitation of this perspective is that the reported studies primarily focus on psychiatric risk CNVs, with minimal discussion of SNVs. This reflects the comparatively limited dimensional research available for SNVs, which will be important to address in future work.

The transition to dimensional approaches has important implications for clinical practice. By providing a more detailed and accurate characterisation of neurodevelopmental and neuropsychiatric phenotypes, these frameworks can facilitate early identification of at-risk individuals and enable the implementation of preventive measures.

Despite the promise of dimensional frameworks, several challenges remain. Current instruments often fail to capture the full range of relevant symptoms. There is also a need for measures that are culturally sensitive and can be used across diverse populations.

In conclusion, the adoption of dimensional frameworks represents a significant step forward in our understanding of neurodevelopmental and neuropsychiatric disorders in children with NGCs. By capturing the full complexity of these conditions, these frameworks can improve diagnostic accuracy, inform personalised treatment strategies, and ultimately enhance outcomes for affected individuals and their families. Continued research and collaboration are essential to fully realise the potential of these approaches and to address the remaining challenges in the field.

## Data Availability

The original contributions presented in the study are included in the article/supplementary material. Further inquiries can be directed to the corresponding author.

## Footnotes

1 Although the lack of precise definitional boundaries, within-category heterogeneity and across category overlaps across NDDs and NPDs are acknowledged, in this paper, we have adopted DSM-5-TR and ICD-11 definition of NDD as behavioral and cognitive disorders that arise during the developmental period, involving significant difficulties in the acquisition and execution of specific intellectual, motor, language, academic, or social functions. Thus, we refer to NDDs as specific diagnostic categories described under the NDD section of the DSM-5-TR and ICD-11. For NPD, we specifically refer to the following existing diagnostic categories: schizophrenia spectrum and other psychiatric disorders, bipolar and related disorders, depressive and anxiety disorders, obsessive-compulsive and related disorders, sleep-wake disorders, disruptive, impulse-control, and conduct disorders, and personality disorders.
